# Defibrillation strategies for refractory ventricular fibrillation out‐of‐hospital cardiac arrest: A systematic review and network meta‐analysis

**DOI:** 10.1111/anec.13075

**Published:** 2023-07-22

**Authors:** Mohamed T. Abuelazm, Ahmed Ghanem, Basant E. Katamesh, Abdul Rhman Hassan, Hassan Abdalshafy, Amith Reddy Seri, Ahmed K. Awad, Mohamed Abdelnabi, Basel Abdelazeem

**Affiliations:** ^1^ Faculty of Medicine Tanta University Tanta Egypt; ^2^ Cardiology Department The Lundquist Institute Torrance California USA; ^3^ Faculty of Medicine Cairo University Cairo Egypt; ^4^ Department of Internal Medicine McLaren Health Care Flint Michigan USA; ^5^ Department of Internal Medicine Michigan State University East Lansing Michigan USA; ^6^ Faculty of Medicine Ain‐Shams University Cairo Egypt; ^7^ Department of Clinical Pharmacy University of Michigan Ann Arbor Michigan USA

**Keywords:** cardiac arrest, meta‐analysis, OHCA, resuscitation, systematic review, ventricular fibrillation

## Abstract

**Background and Objective:**

Double sequential external defibrillation (DSED) and vector‐change defibrillation (VCD) have been suggested to enhance clinical outcomes for patients with ventricular fibrillation (VF) refractory of standard defibrillation (SD). Therefore, this network meta‐analysis aims to evaluate the comparative efficacy of DSED, VCD, and SD for refractory VF.

**Methods:**

A systematic review and network meta‐analysis synthesizing randomized controlled trials (RCTs) and comparative observational studies retrieved from PubMed, EMBASE, WOS, SCOPUS, and Cochrane through November 15th, 2022. R software netmeta and netrank package (R version 4.2.0) and meta‐insight software were used to pool dichotomous outcomes using odds ratio (OR) presented with the corresponding confidence interval (CI). Our protocol was prospectively published in PROSPERO with ID: CRD42022378533.

**Results:**

We included seven studies with a total of 1632 participants. DSED was similar to SD in survival to hospital discharge (OR: 1.14 with 95% CI [0.55, 2.83]), favorable neurological outcome (modified Rankin scale ≤2 or cerebral performance category ≤2) (OR: 1.35 with 95% CI [0.46, 3.99]), and return of spontaneous circulation (ROSC) (OR: 0.81 with 95% CI [0.43; 1.5]). In addition, VCD was similar to SD in survival to hospital discharge (OR: 1.12 with 95% CI [0.27, 4.57]), favorable neurological outcome (OR: 1.01 with 95% CI [0.18, 5.75]), and ROSC (OR: 0.88 with 95% CI [0.24; 3.15]).

**Conclusion:**

Double sequential external defibrillation and VCD were not associated with enhanced outcomes in patients with refractory VF out‐of‐hospital cardiac arrest, compared to SD. However, the current evidence is still inconclusive, warranting further large‐scale RCTs.

## Graphical abstract

Double sequential external defibrillation (DSED) and vector change defibrillation (VCD) were not associated with enhanced outcomes in patients with out‐of‐hospital cardiac arrest (OHCA), compared to standard defibrillation (SD). However, the only complete randomized clinical trial (RCT) so far has shown that DSED could be a promising technique to replace SD for OHCA. Therefore, further large‐scale RCTs with unified outcome definitions and DSED protocol are still warranted before the endorsement of DSED in clinical practice.

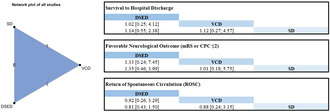

## 1 INTRODUCTION

While cardiovascular disease is the leading cause of death worldwide, out‐of‐hospital cardiac arrest (OHCA) remains a major health crisis. In the United States, around 356,000 people are treated for OHCA annually (McCarthy et al., 2018). In Europe, approximately 300,000 people are treated for OHCA by emergency medical services (EMS) annually (Zeppenfeld et al., 2022). The outcome of the OHCA varies in different communities for different reasons, including variations in the resuscitation process, awareness of the public of basic life support and their access to automated external defibrillation (AED), the readiness of EMS, and post‐hospitalization care (Abrams et al., 2013; Sorensen, 2015; Zive et al., 2011). The survival rate of the EMS‐treated OHCA was as low as 10.6% according to the cardiac arrest registry to enhance the survival (CARE) registry of 2019 (Virani et al., 2021).

According to the American Heart Association (AHA) and the European Society of Cardiology (ESC), initiation of high‐quality cardiopulmonary resuscitation (CPR) and application of AED by bystanders are class I recommendations as they increase the neurologically intact survival (NIS) of OHCA by multiple folds (Berger, 2017; Zeppenfeld et al., 2022). Patients who present with shockable rhythm, such as ventricular fibrillation (VF) or pulseless ventricular tachycardia (pVT) and patients with return of spontaneous circulation (ROSC) in the field have higher NIS rates compared to those with non‐shockable rhythms (Daya et al., 2015; McCarthy et al., 2018; Okubo et al., 2017; Sasson et al., 2010). Besides basic life support, EMS uses antiarrhythmic medications and defibrillators to treat VF and pVT. Most VF patients respond to treatment; however, there is a subset of patients who do not respond to either defibrillation or antiarrhythmic medications (Eifling et al., 2011; Sakai et al., 2010). Refractory VF (RVF) is identified as a VF that does not resolve after three or five consecutive defibrillation attempts in addition to antiarrhythmic medications (Emmerson et al., 2017; Leacock, 2014; Miraglia et al., 2020). With every defibrillation attempt, the success rate of terminating the VF decreases, and the mortality rate increases (Eifling et al., 2011; Koster et al., 2008). Exploring other means to treat RVF led to the development of different techniques of defibrillation.

Double sequential external defibrillators (DSED) and vector‐change defibrillators (VCD) have been suggested for the treatment of RVF in OHCA due to the growing evidence of their efficacy (Beck et al., 2019; Cheskes et al., 2019, 2020, 2022; Emmerson et al., 2017; Kim et al., 2020; Mapp et al., 2019; Ross et al., 2016). In standard defibrillation (SD), one pad is placed anterior to the chest wall, while the other one is placed on the lateral chest wall to deliver the shock. In the VCD, the pads' position is anterior–posterior instead of anterior‐lateral. Meanwhile, the DSED uses two defibrillators with two sets of pads, one is placed in the anterior‐lateral position, while the other is placed in the anterior–posterior position. The two shocks generated from the DSED are delivered either at the same time or sequentially to one another. The exact mechanism by which the DSED terminates the RVT is unknown. However, its efficacy is reasoned to different theories, including higher energy delivery to overcome the increased defibrillatory threshold, exposing more myocytes to the shock, and lowering the defibrillation threshold (Miraglia et al., 2020).

With the lack of randomized control trials (RCTs) to evaluate the efficacy of the new defibrillation techniques and the available evidence is mainly based on observational data (Deakin et al., 2020; Delorenzo et al., 2019; Miraglia et al., 2020), there is a need to explore their efficacy compared to SD in the OHCA. After the publication of the first RCT comparing DSED, VCT, and SD, we thought of conducting this network meta‐analysis to evaluate the comparative efficacy of DSED, VCD, and SD in RVT in OHCA.

## 2 METHODOLOGY

### 2.1 Protocol registration

We submitted this systematic review and meta‐analysis' protocol to PROSPERO with ID: CRD42022378533. Preferred Reporting Items for Systematic Reviews and Meta‐Analysis (PRISMA) extension statement for network meta‐analyses (Hutton et al., 2015) and the Cochrane Handbook of Systematic Reviews and Meta‐analysis (Higgins et al., 2019) were strictly followed during this study's conduction.

### 2.2 Data sources and search strategy

Two reviewers (B.A. and M.T.A.) searched PubMed (MEDLINE), Web of Science, Cochrane, SCOPUS, and EMBASE up to November 15th, 2022, without using any search limits or filters. The search strategy for each database is outlined in (Table S1).

### 2.3 Eligibility criteria

We included studies fulfilling the following criteria: RCTs or observational comparative studies recruiting patients with RVF who had experienced OHCA and underwent either DSED controlled by VCD or SD. Our primary outcome was survival to hospital discharge. Our secondary outcomes were favorable neurological recovery measured by either a modified Rankin Scale (mRS) or Cerebral Performance Category (CPC) ≤2, ROSC, and survival to hospital discharge. Review articles, letters, single‐arm clinical trials, animal studies, case series, case reports, comments, and consensus documents were excluded from this systematic review and meta‐analysis.

### 2.4 Study selection

Using the previous eligibility criteria, three independent reviews (B.E.K., A.R.H., and H.A.) initiated the titles and abstract screening after excluding duplicates via Covidence online software. Then (B.E.K., A.R.H., and H.A.) proceeded with the full‐text screening. Disagreements were resolved by discussion or inviting (B.A.) to reach a consensus.

### 2.5 Data extraction

Four independent reviewers (B.E.K., A.R.H., H.A., and A.R.S.) extracted the following using an Excel data extraction form: summary characteristics (first author name, year of publication, country, study design, total participants, DSED indication, DSED technique details, primary outcome, and follow‐up duration); baseline characteristics (age, sex, number of patients in each arm, emergency medical services (EMS) witnessed arrest, bystander cardiopulmonary resuscitation, and time to response); and outcomes data. Disagreements were resolved through discussion.

### 2.6 Risk of bias and quality assessment

Four independent reviewers (B.E.K., A.R.H., H.A., and A.R.S.) used Risk of Bias In Non‐Randomized Studies ‐ of Interventions (ROBINS‐I) (Sterne et al., 2016) to evaluate the quality of observational studies and Cochrane updated risk of bias (ROB 2) (Sterne et al., 2019) to evaluate the quality of RCTs. ROBINS‐I consists of seven items: bias due to confounding, bias in the selection of participants into the study, bias in classification of interventions, bias due to deviations from intended interventions, bias due to missing data, bias in the measurement of outcomes, and bias in the selection of the reported result. While ROB 2 consists of five items: randomization process, deviations from intended interventions, missing outcome data, measurement of the outcome, and selection of the reported result. Conflicts were discussed and resolved by consensus.

### 2.7 Statistical analysis

We conducted a network meta‐analysis using a frequentist framework (Hutton et al., 2015), pooling dichotomous outcomes using odds ratio (OR) presented with the corresponding 95% confidence interval (CI). Analysis was performed using the R‐software netmeta and netrank package (R version 4.2.0) and meta‐insight software (Owen et al., 2019; R Core Team, 2021; Rücker et al., 2016) with statistical inconsistency between network arms and was evaluated by calculating *I* ^2^.

Revman version 5.4 (Cochrane Training, 2021) was used to pool survival to hospital admission using OR presented with the corresponding 95% CI using the random‐effect model. *I* ^2^ and chi‐squared tests were used to evaluate the statistical heterogeneity. *p*‐value < .05 was considered significant for the chi‐squared test, and *I* ^2^ > 50% indicated substantial heterogeneity, in which case sensitivity analysis was conducted by excluding one study each time to determine the source of heterogeneity. Finally, we did not investigate the publication bias by funnel plots as we included less than 10 studies (Egger et al., 1997).

## 3 RESULTS

### 3.1 Search results and study selection

**FIGURE 1:**
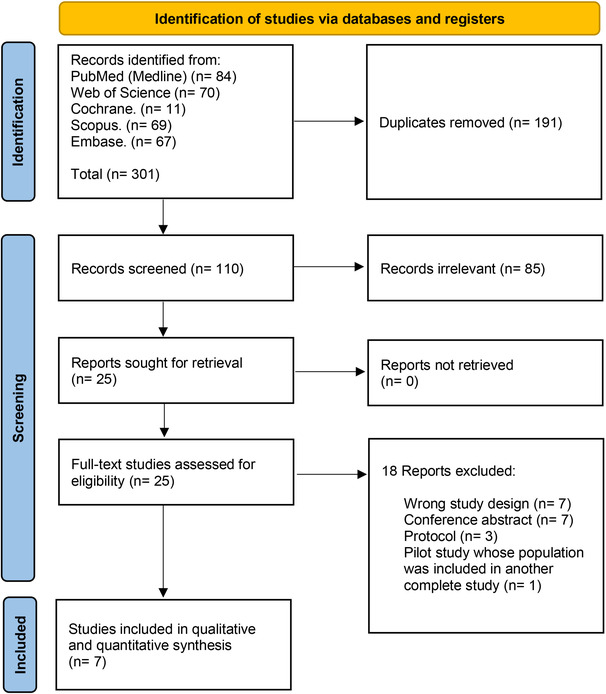
PRISMA flow chart of the screening process.

We initially identified a total of 301 records after searching the databases. One hundred and ninety‐one duplicates were removed using Covidence, which left 110 records for title and abstract screening. We excluded 85 records and finally screened 25 full‐text articles to include six observational studies and an RCT (Figure 1).

### 3.2 Characteristics of included studies

We included seven studies with a total of 1632 participants (Beck et al., 2019; Cheskes et al., 2019, 2022; Emmerson et al., 2017; Kim et al., 2020; Mapp et al., 2019; Ross et al., 2016). Seven studies used DSED and SD (Beck et al., 2019; Cheskes et al., 2019, 2022; Emmerson et al., 2017; Kim et al., 2020; Mapp et al., 2019; Ross et al., 2016), while only Cheskes et al. (2022) used VCD. Further summary characteristics of the included studies are outlined in (Table 1). Moreover, baseline data for the included participants are outlined in (Table 2).

**TABLE 1:** Summary characteristics of the included studies.

Study ID	Study design	Country	Total participants (*N*)	DSED indication	DSED technique details			Primary outcomes
					Pad placement	Mean shock number	Joules	
Beck et al. (2019)	Retrospective observational	USA	310	VF refractory to 3 or more sequential defibrillation attempts	AP or AL	2.2	720	Prehospital ROSC, survival to hospital admission, survival to 72 h, and survival to hospital discharge
Cheskes et al. (2019)	Retrospective observational	Canada	252	VF refractory to three or more sequential defibrillation attempts	AP	2.3	400	VF termination and ROSC
Cheskes et al. (2022)	Cluster RCT with crossover	Canada	405	VF refractory to three or more sequential defibrillation attempts	AP	N/A	400 or 720	Survival to hospital discharge
Emmerson et al. (2017)	Retrospective observational	UK	220	VF refractory to six or more sequential defibrillation attempts	AP or AL	2.5	720	Pre‐hospital ROSC, ROSC sustained to hospital admission and survival to hospital discharge
Kim et al. (2020)	Retrospective observational	South Korea	38	VF refractory to three or more sequential defibrillation attempts	AP or AL	7 (median)	400	Survival to hospital discharge
Mapp et al. (2019)	Matched case–control	USA	128	VF refractory to three or more sequential defibrillation attempts	AP or AL	N/A	400	Survival to hospital admission
Ross et al. (2016)	Retrospective cohort	USA	279	Recurrent and refractory ventricular fibrillation	AP or AL	N/A	400	Neurologically intact survival to hospital discharge

Abbreviations: AL, anterolateral; AP, anteroposterior; DSED, double sequential external defibrillation; *N*, number; N/A, not available; RCT, randomized controlled trial; ROSC, return of spontaneous circulation; UK, United Kingdom; USA, United States of America; VF, ventricular fibrillation.

**TABLE 2:** Baseline characteristics of the participants.

Study ID	The number of patients (*N*)		Age (years), mean (SD)		Gender (male), *N* (%)		EMS witnessed arrest, *N* (%)		Bystander CPR, *N* (%)		Time to response	
	DSED or VCD	Standard	DSED or VCD	Standard	DSED or VCD	Standard	DSED or VCD	Standard	DSED or VCD	Standard	DSED or VCD	Standard
Beck et al. (2019)	71	239	62.2 (14.1)	62.3 (14.3)	61 (84.5)	174 (72.80)	4 (5.6)	20 (8.4)	52 (73.2)	149 (62.3)	5.6 (2.0)	5.5 (2.2)
Cheskes et al. (2019)	51	201	61.8 (14.3)	63.8 (15.7)	43 (84.3)	170 (84.6)	2 (3.9)	5 (2.5)	34 (68)	109 (55.1)	7.1 (2.9)	7.1 (2.8)
Cheskes et al. (2022)	VCD (*N* = 144) DSED (*N* = 125)	136	VCD 63.8 (13.2), DSED 63.0 (16.8)	64.0 (14.4)	VCD 127 (88.2), DSED 106 (84.8)	109 (80.1)	N/A	N/A	VCD 90 (62.5), DSED 71 (56.8)	74 (54.4)	VCD 7.4 (6.9–9.0), DSED 7.8 (6.0–9.4)	7.4 (5.7–9.9)
Emmerson et al. (2017)	45	175	59.8 (13.8)	62.5 (16.5)	42 (93.3)	144 (82.3)	4 (8.9)	13 (7.4)	32 (71.1)	115 (65.7)	N/A	N/A
Kim et al. (2020)	17	21	56.92 (18.09)	62.24 (19.84)	14 (82.4)	17 (81.0)	NA	NA	10 (58.8)	9 (42.9)	8.5 (6.8–11)	7 (4–10)
Mapp et al. (2019)	25	103	58.3 (10.6)	58.4 (13.3)	22 (88)	80 (77.7)	2 (8)	2 (1.9)	8 (32)	52 (50.5)	8 (6–12)	8 (6–10)
Ross et al. (2016)	50	229	N/A	N/A	50 (76)	168 (73.4)	19 (38)	125 (54.6)	15 (30)	104 (45.4)	N/A	N/A

Abbreviations: DSED, double sequential external defibrillation; *N*, number; N/A, not available; SD, standard deviation; VCD, vector change defibrillation.

### 3.3 Risk of bias and quality of evidence

**FIGURE 2:**
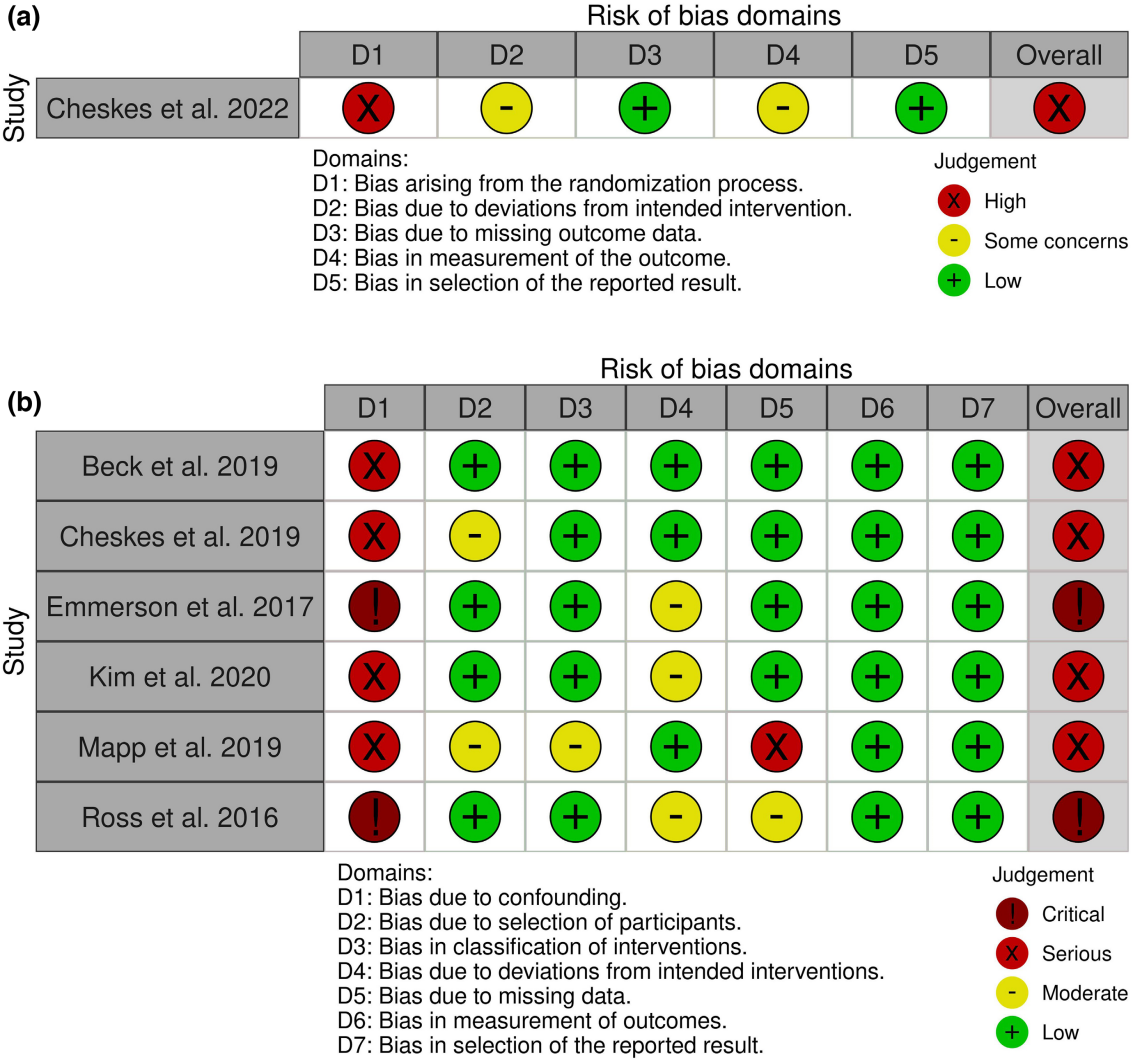
Quality assessment of the included studies: ((a) RCTs assessed by ROB‐2, (b) observational studies assessed by ROBINS‐I).

Cheskes et al. (2022) was associated with a high risk of selection bias as paramedics were aware of the allocated intervention besides SD to make required preparations. In addition, it was associated with some concerns regarding deviations from intended interventions and outcome assessment (Figure 2a). Observational studies were mainly associated with a serious to critical risk of confounding due to a lack of adjusting for covariates. In addition, there was a moderate risk of bias due to deviations from the intended interventions in three studies, selection bias in two studies, and due to missing data in one study (Figure 2b).

### 3.4 Primary outcome: survival to hospital discharge

**FIGURE 3:**
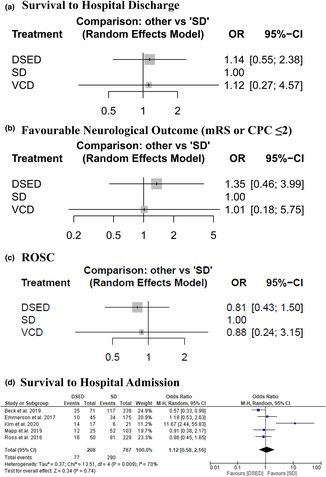
Forest plot of the efficacy outcomes. CI, confidence interval; OR, odds ratio. (a) Survival to Hospital Discharge, (b) Favourable Neurological Outcome (mRS or CPC ≤2), (c) ROSC, and (d) Survival to Hospital Admission.

Compared to SD, neither DSED (OR: 1.14 with 95% CI [0.55; 2.38]) nor VCD (OR: 1.12 with 95% CI [0.27; 4.57]) improved survival to discharge. There was also no difference between DSED and VCD (OR: 1.02 with 95% CI [0.25; 4.12]) (Table 3, Figure 3a, Figures S1 and S2). Our analysis showed a substantial heterogeneity (*I* ^2^ = 55%, *p* = .04).

**TABLE 3:** Ranking table for network meta‐analysis outcomes.

**Survival to hospital discharge**		
DSED	1.58 [0.33; 7.44]	1.14 [0.55; 2.38]
1.02 [0.25; 4.12]	VCD	1.80 [0.37; 8.76]
1.14 [0.55; 2.38]	1.12 [0.27; 4.57]	SD
**Favorable neurological outcome (mRS or CPC ≤2)**		
DSED	1.95 [0.30; 12.83]	1.35 [0.46; 3.99]
1.33 [0.24; 7.45]	VCD	1.53 [0.23; 10.42]
1.35 [0.46; 3.99]	1.01 [0.18; 5.75]	SD
**ROSC**		
DSED	1.58 [0.38; 6.59]	0.81 [0.43; 1.50]
0.92 [0.26; 3.29]	VCD	1.52 [0.36; 6.41]
0.81 [0.43; 1.50]	0.88 [0.24; 3.15]	SD

### 3.5 Secondary outcomes

#### 3.5.1 Favorable neurological outcome (mRS or CPC ≤2)

Compared to SD, neither DSED (OR: 1.35 with 95% CI [0.46; 3.99]) nor VCD (OR: 1.01 with 95% CI [0.18; 5.75]) improved neurological recovery. There was also no difference between DSED and VCD (OR: 1.33 with 95% CI [0.24; 7.45]) (Table 3, Figure 3b, Figures S3 and S4). Our analysis showed low heterogeneity (*I* ^2^ = 22%, *p* = .32).

#### 3.5.2 ROSC

Compared to SD, neither DSED (OR: 0.81 with 95% CI [0.43; 1.5]) nor VCD (OR: 0.88 with 95% CI [0.24; 3.15]) improved the ROSC rate. There was also no difference between DSED and VCD (OR: 0.92 with 95% CI [0.26; 3.29]) (Table 3, Figure 3c, Figures S5 and S6). Our analysis showed a substantial heterogeneity (*I* ^2^ = 72%, *p* = .03).

#### 3.5.3 Survival to hospital admission

There was no difference between DSED and SD (OR: 1.12 with 95% CI [0.58, 2.16]) (Figure 3d). Our results were heterogeneous (*I* ^2^ = 70%, *p* = .009). We conducted a sensitivity analysis to investigate the source of heterogeneity, and it was best resolved after excluding Kim et al. (2020) (*I* ^2^ = 0%, *p* = .47) (Table S2). After excluding Kim et al. (2020), there was also no difference between DSED and SD (OR: 0.78 with 95% CI [0.56, 1.10]).

## 4 DISCUSSION

Based on our pooled analysis, substituting SD with DSED or VCD did not improve survival to hospital discharge, favorable neurological outcomes, ROSC, and survival to hospital admission compared to continuing SD. RVF is an unfavorable health issue that affects OHCA outcomes negatively compared to other shockable rhythms. Recently, new data emerged suggesting the superiority of DSED and VCD over SD in suppressing the RVF and improving its outcome (Cheskes et al., 2022). We conducted this systematic review and meta‐analysis to synthesize the latest data available about the efficacy of DSED and VCD. Accordingly, we identified seven studies with a total of 1632 patients. The included studies lacked a unified protocol, and their sample size was relatively small. All the included patients received the recommended standard of care, including chest compression and anti‐arrhythmic medications, before starting SD.

In the only performed RCT, Cheskes et al. (2022) reported that applying DSED and VCD significantly improved the survival to hospital discharge, compared to SD (30.4% and 21.7% vs. 13.3%), consequently. On the other hand, none of the other included observational studies reported improvement in survival to hospital discharge (Beck et al., 2019; Cheskes et al., 2019; Emmerson et al., 2017; Kim et al., 2020; Mapp et al., 2019; Ross et al., 2016). Our analysis was mainly weighted by observational studies, which were associated with a serious to critical overall risk of bias, which may significantly affect our findings, given that only Cheskes et al. (2022) showed a significant effect of DSED and VCD over SD. Accordingly, further RCTs are still required to confirm this effect. Moreover, Cheskes et al. (2022) also reported that applying DSED but not VCD was associated with more patients with favorable neurological outcomes, compared to SD (27.4% vs. 11.2% consequently). However, none of the included observational studies reported similar results, and neither did our pooled analysis.

Kim et al. (2020) reported an increase in survival to hospital admission with DSED, compared to SD. However, their study was conducted in the emergency department (Kim et al., 2020). They also reported a significant difference in the witnessed arrest and bystander CPR between the DSED and SD (Kim et al., 2020), which might be a confounder for their results and accordingly the reason behind heterogeneity as it was resolved after excluding Kim et al. (2020).

Terminating the RVF is challenging, and the success rate decreases with every defibrillation attempt (Cheskes et al., 2019; Ideker et al., 1991). As mentioned earlier, DSED and VCD were thought to be effective in terminating the RVF for different reasons, as follows. A stronger electrical current can overcome and reduce a higher threshold of fibrillation (Jones et al., 1988). In addition, more victors deliver larger energy currents, overcoming the increased body weight in obese patients (Zhang et al., 2002), and defibrillating myocardial cells from different directions (Kerber et al., 1994; Merlin et al., 2016). With most of the reported successful attempts to terminate the RVF using DSED or VCD being case reports, there are no clear guidelines on how to use them. However, it was noticed by Cheskes et al. (2020) that the success of DSED in terminating the RVF was time‐dependent. With the lack of difference between the SD and DSED overall in terminating the RVF, the VF termination rate with early attempts (4–8 attempts) and late attempts (9–17 attempts) was higher with DSED. Cheskes et al. (2022) also reported that both DSED and VCD were superior to SD in terminating the RVF, with DSED being better than VCD.

Return of spontaneous circulation before hospital admission is a very important factor in predicting the outcome of OHCA. Patients who experience ROSC have a higher survival rate (Wampler et al., 2012). Beck et al. (2019) reported that patients who received DSED had lower ROSC than those who received only SD. However, they reported that patients who received DSED also received a higher number of shocks, compared to those in the SD group, which may be due to the delay in receiving the DSED. This is supported by the findings of Cheskes et al. (2020) who reported higher ROSC with DSED in a time‐dependent manner. When the DSED was used early with a median of 4 prior SD attempts, it improved the ROSC. On the other hand, when it was used late with a median of 7 prior SD attempts, it had a similar effect on the ROSC as the SD. Emmerson et al. (2017) and Ross et al. (2016) also reported similar ROSC with the use of both DSED and SD. Cheskes et al. (2022) also reported in their most recent RCT (Cheskes et al., 2019) that the use of DSED and not the VCD increased the ROSC significantly, compared to the SD alone. However, we found no benefits of using DSED or VCD on ROSC.

### 4.1 Limitations

Our review has a few limitations. First, we included a small number of studies with only one RCT, which makes our pooled analysis vulnerable to various confounding variables. Second, all of the studies showed a serious to critical overall risk of bias. Third, only one study used the VCD arm, which can make our data regarding VCD seriously underpowered. Finally, some of our outcomes showed substantial heterogeneity due to the differences between the included studies, especially regarding the DSED protocol. Accordingly, the results of our pooled analysis must be interpreted with caution.

### 4.2 Implications for future research

The lack of a uniform definition of RVF and uniform protocol of when to use DSED or VCD in RVF led to differences in the timing and the number of SD attempts before starting DSED/VCD. As mentioned earlier, the time when DSED/VCD is being used might have a role in determining its efficacy on different outcomes. Given the time difference before starting DSED/VCD in the included studies, there is a possibility for resuscitation time bias. The amount of energy used in the included studies was different. The number of witnessed arrests and the number of patients who received bystander CPR was higher in the SD group compared to the DSED/VCD groups. It is known that both witnessed arrests and bystanders' CPR‐receiving arrests have better outcomes. This makes it hard to get a conclusion about the efficacy of DSED. Therefore, future RCTs are required to better adjust the previously mentioned confounding variables. Furthermore, there are no reported safety issues from using higher energy amounts in either DSED or VCD; however, with the lack of long‐term RCTs, the exact safety profile cannot be concluded. This warrants that future RCTs should measure and follow up on the adverse effects of both DSED and VCD over a longer follow‐up duration to better understand the safety profile of both techniques.

## 5 CONCLUSION

Double sequential external defibrillation and VCD were not associated with enhanced outcomes in patients with RVF out‐of‐hospital cardiac arrest, compared to SD. However, the current evidence is still not conclusive, depending mainly on observational studies. However, the only complete RCT, so far, showed that DSED could be a promising technique to replace SD for RVF. Therefore, further large‐scale RCTs with unified outcome definitions and DSED protocol are still warranted before the endorsement of DSED in clinical practice.

## AUTHOR CONTRIBUTIONS

Mohamed T. Abuelazm conceived the idea. Basel Abdelazeem and Mohamed T. Abuelazm designed the research workflow. Basel Abdelazeem and Mohamed T. Abuelazm searched the databases. Basant E. Katamesh, Abdul Rhman Hassan, Hassan Abdalshafy, and Amith Reddy Seri screened the retrieved records, extracted relevant data, assessed the quality of evidence, and Basel Abdelazeem resolved the conflicts. Mohamed T. Abuelazm and Ahmed K. Awad performed the analysis. Ahmed Ghanem, Mohamed Abdelnabi, and Mohamed T. Abuelazm wrote the final manuscript. Basel Abdelazeem and Mohamed Abdelnabi supervised the project. All authors have read and agreed to the final version of the manuscript.

## CONFLICT OF INTEREST STATEMENT

The authors declare no conflicts of interest.

## ETHICS STATEMENT

None declared.

## Supporting information


Appendix S1

## Data Availability

Data are available upon reasonable request.
