# Supplementary material for: Defibrillation strategies for refractory ventricular fibrillation out‐of‐hospital cardiac arrest: A systematic review and network meta‐analysis
Source: Ann Noninvasive Electrocardiol. 2023 Jul 22;28(5):e13075. doi: 10.1111/anec.13075 (PMC10475889; doi:10.1111/anec.13075)
Supplement: Supplementary file 1 — Appendix S1 [file ANEC-28-e13075-s001.docx]

**Supplementary material:**

**Title.**

**Defibrillation Strategies for Refractory Ventricular Fibrillation Out-of-Hospital Cardiac Arrest: A Systematic Review and Network Meta-Analysis.**

**Running Title.**

**DSED vs, VCD vs. SD for Refractory OHCA.**

**Authors.**

Mohamed T. Abuelazm^1,*^, Ahmed Ghanem^2,*^, Basant E. Katamesh^1^, Abdul Rhman Hassan^1^, Hassan Abdalshafy^3^, Amith Reddy Seri^4,5^, Ahmed K. Awad^6^, Mohamed Abdelnabi^7^, Basel Abdelazeem^4,5^.

**Affiliations.**

1. Faculty of Medicine, Tanta University, Tanta, Egypt.
2. Cardiology Department, The Lundquist Institute, Torrance, CA, USA.
3. Faculty of Medicine, Cairo University, Cairo, Egypt.
4. Department of Internal Medicine, McLaren Health Care, Flint, Michigan, USA.
5. Department of Internal Medicine, Michigan State University, East Lansing, Michigan, USA.
6. Faculty of Medicine, Ain-Shams University, Cairo, Egypt.
7. Department of Clinical Pharmacy, University of Michigan, Ann Arbor, Michigan, USA.

*, Both authors contributed equally to this work and are co-first authors.

**Keywords.**

Resuscitation, cardiac arrest, OHCA, ventricular fibrillation, systematic review, meta-analysis.

**Contents:**

**Tables.**

Table S1: Search terms and results in different databases.

Table S2: Sensitivity analysis for pair-wise meta-analysis.

**Figures.**

Figure S1: Network plot of survival to hospital discharge.

Figure S2: Forest plot of individual study results grouped by treatment component for survival to hospital discharge.

Figure S3: Network plot of favourable Neurological Outcome (mRS or CPC ≤2).

Figure S4: Forest plot of individual study results grouped by treatment component for favourable Neurological Outcome (mRS or CPC ≤2).

Figure S5: Network plot of ROSC.

Figure S6: Forest plot of individual study results grouped by treatment component for ROSC.

| Database | Search Terms | Search Field | Search Results |
| --- | --- | --- | --- |
| PubMed | ("refractory ventricular fibrillation" OR "cardiac arrest" OR "recurrent ventricular fibrillation" OR RVF OR "ventricular fibrillation" OR VF OR "shockable rhythms" OR "pre-hospital cardiac arrest" OR "out-of-hospital cardiac arrest" OR "heart arrest" OR "OHCA" or "in-hospital cardiac arrest" OR IHCA) AND ("double sequential external defibrillation" OR DSED OR "double sequential defibrillation" OR "double defibrillation" OR "dual defibrillation" OR "dual shock" OR "double simultaneous defibrillation" OR "dual simultaneous defibrillation" OR "double sequential defibrillation" OR "dual sequential defibrillation" OR DSiD OR DSD OR "vector change") | All Field | 84 |
| Cochrane | ("refractory ventricular fibrillation" OR "cardiac arrest" OR "recurrent ventricular fibrillation" OR RVF OR "ventricular fibrillation" OR VF OR "shockable rhythms" OR "pre-hospital cardiac arrest" OR "out-of-hospital cardiac arrest" OR "heart arrest" OR "OHCA" or "in-hospital cardiac arrest" OR IHCA) AND ("double sequential external defibrillation" OR DSED OR "double sequential defibrillation" OR "double defibrillation" OR "dual defibrillation" OR "dual shock" OR "double simultaneous defibrillation" OR "dual simultaneous defibrillation" OR "double sequential defibrillation" OR "dual sequential defibrillation" OR DSiD OR DSD OR "vector change") | All Field | 11 |
| WOS | ("refractory ventricular fibrillation" OR "cardiac arrest" OR "recurrent ventricular fibrillation" OR RVF OR "ventricular fibrillation" OR VF OR "shockable rhythms" OR "pre-hospital cardiac arrest" OR "out-of-hospital cardiac arrest" OR "heart arrest" OR "OHCA" or "in-hospital cardiac arrest" OR IHCA) AND ("double sequential external defibrillation" OR DSED OR "double sequential defibrillation" OR "double defibrillation" OR "dual defibrillation" OR "dual shock" OR "double simultaneous defibrillation" OR "dual simultaneous defibrillation" OR "double sequential defibrillation" OR "dual sequential defibrillation" OR DSiD OR DSD OR "vector change") | All Field | 70 |
| SCOPUS | TITLE-ABS-KEY ( ( "refractory ventricular fibrillation" OR "cardiac arrest" OR "recurrent ventricular fibrillation" OR rvf OR "ventricular fibrillation" OR vf OR "shockable rhythms" OR "pre-hospital cardiac arrest" OR "out-of-hospital cardiac arrest" OR "heart arrest" OR "OHCA" OR "in-hospital cardiac arrest" OR ihca ) AND ( "double sequential external defibrillation" OR dsed OR "double sequential defibrillation" OR "double defibrillation" OR "dual defibrillation" OR "dual shock" OR "double simultaneous defibrillation" OR "dual simultaneous defibrillation" OR "double sequential defibrillation" OR "dual sequential defibrillation" OR dsid OR dsd OR "vector change" ) ) | Title, Abstract, Keywords | 69 |
| EMBASE | #3. #1 AND #2  #2. 'double sequential external  defibrillation':ti,ab,kw OR dsed:ti,ab,kw OR  'double defibrillation':ti,ab,kw OR 'dual  defibrillation':ti,ab,kw OR 'dual shock':ti,ab,kw  OR 'double simultaneous defibrillation':ti,ab,kw  OR 'dual simultaneous defibrillation':ti,ab,kw OR  'double sequential defibrillation':ti,ab,kw OR  'dual sequential defibrillation':ti,ab,kw OR  dsid:ti,ab,kw OR dsd:ti,ab,kw OR 'vector  change':ti,ab,kw  #1. 'refractory ventricular fibrillation':ti,ab,kw OR  'cardiac arrest':ti,ab,kw OR 'recurrent  ventricular fibrillation':ti,ab,kw OR  rvf:ti,ab,kw OR 'ventricular  fibrillation':ti,ab,kw OR vf:ti,ab,kw OR  'shockable rhythms':ti,ab,kw OR 'pre-hospital  cardiac arrest':ti,ab,kw OR 'out-of-hospital  cardiac arrest':ti,ab,kw OR 'heart  arrest':ti,ab,kw OR ohca:ti,ab,kw OR 'in-hospital  cardiac arrest':ti,ab,kw OR ihca:ti,ab,kw | All Field | 67 |

Table S1: Search terms and results in different databases.

| Outcome | No. of  participants (DSED/SD) | No. of  trials | Quantitative data synthesis | | | | Heterogeneity analysis | | |
| --- | --- | --- | --- | --- | --- | --- | --- | --- | --- |
|  |  |  | OR | 95% CI | Z value | p-value | df | p-value | I2 (%) |
| **Survival to Hospital Admission** | | | | | | | | | |
| All Studies | 208/767 | 5 | 1.12 | [0.58, 2.16] | 0.34 | 0.74 | 4 | 0.009 | 70 |
| Omitting  Beck et al. 2019 | 137/528 | 4 | 1.43 | [0.64, 3.18] | 0.87 | 0.38 | 3 | 0.02 | 68 |
| Omitting  Emmerson et al. 2017 | 163/592 | 4 | 1.16 | [0.50, 2.68] | 0.34 | 0.73 | 3 | 0.005 | 77 |
| Omitting  Kim et al. 2020 | 191/746 | 4 | 0.78 | [0.56, 1.10] | 1.41 | 0.16 | 3 | 0.47 | 0 |
| Omitting  Mapp et al. 2019 | 183/664 | 4 | 1.24 | [0.54, 2.86] | 0.50 | 0.61 | 3 | 0.004 | 78 |
| Omitting | 158/538 | 4 | 1.30 | [0.52, 3.25] | 0.57 | 0.57 | 3 | 0.004 | 78 |

Table S2: Sensitivity analysis for pair-wise meta-analysis.

*
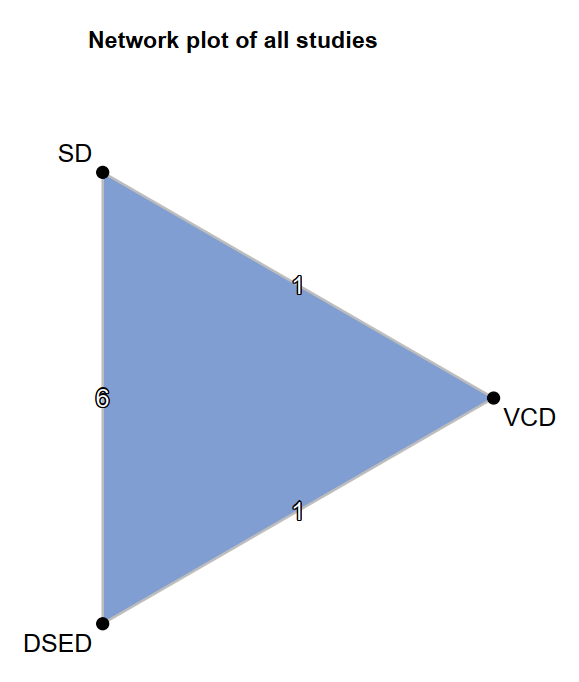
*

Figure S1: Network plot of survival to hospital discharge.

*
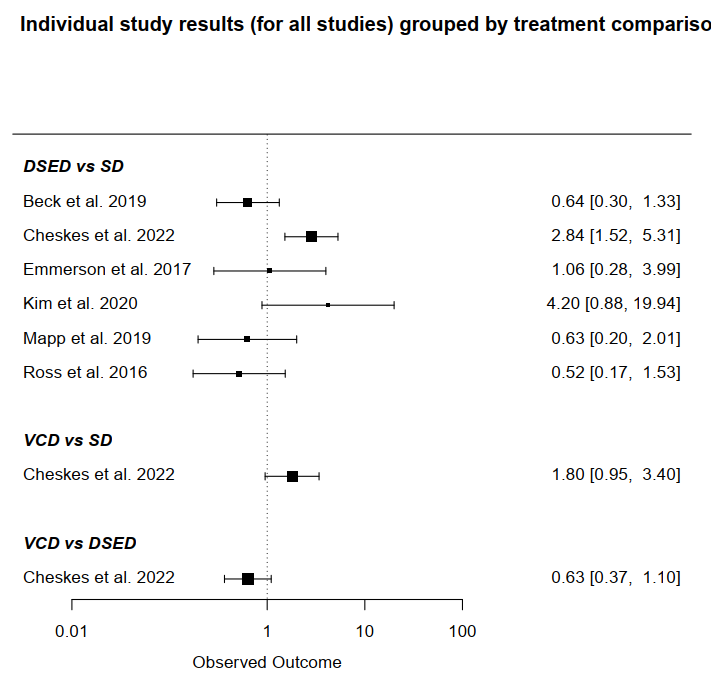
*

Figure S2: Forest plot of individual study results grouped by treatment component for survival to hospital discharge.

*
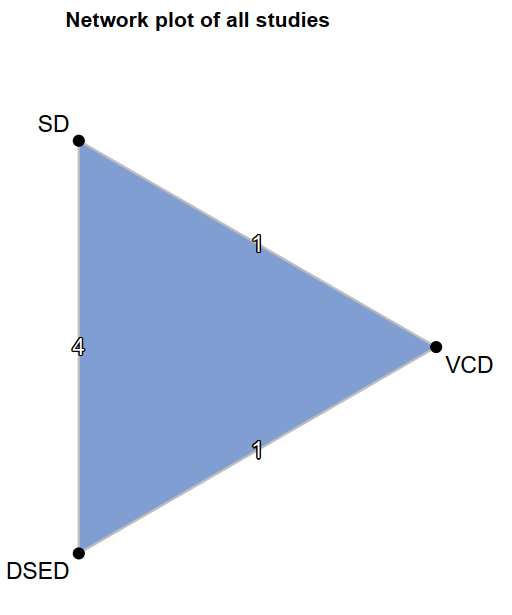
*

Figure S3: Network plot of favourable Neurological Outcome (mRS or CPC ≤2).


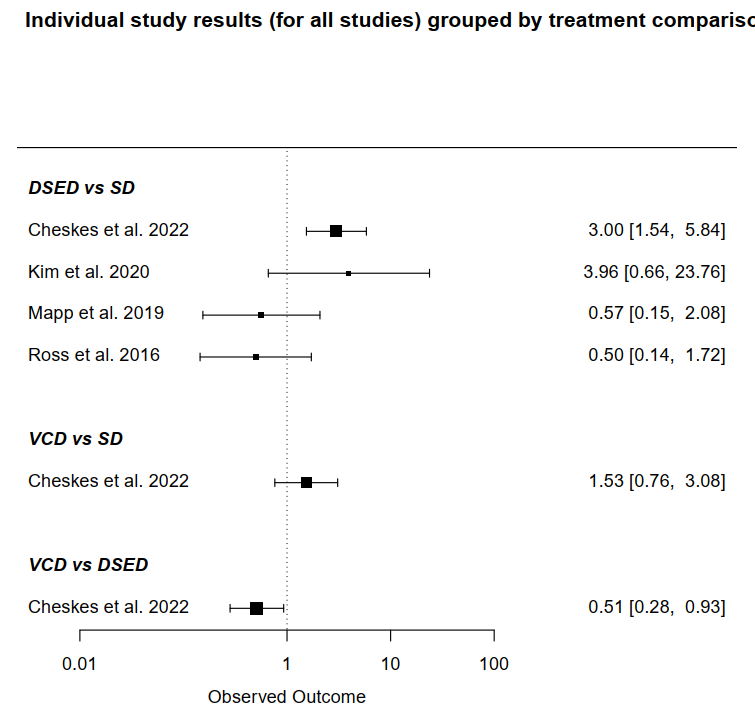


Figure S4: Forest plot of individual study results grouped by treatment component for favourable Neurological Outcome (mRS or CPC ≤2).

*
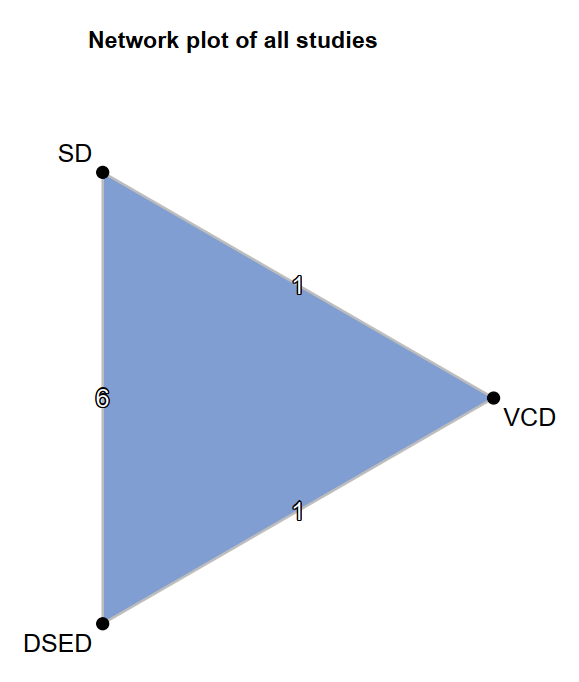
*

Figure S5: Network plot of ROSC.


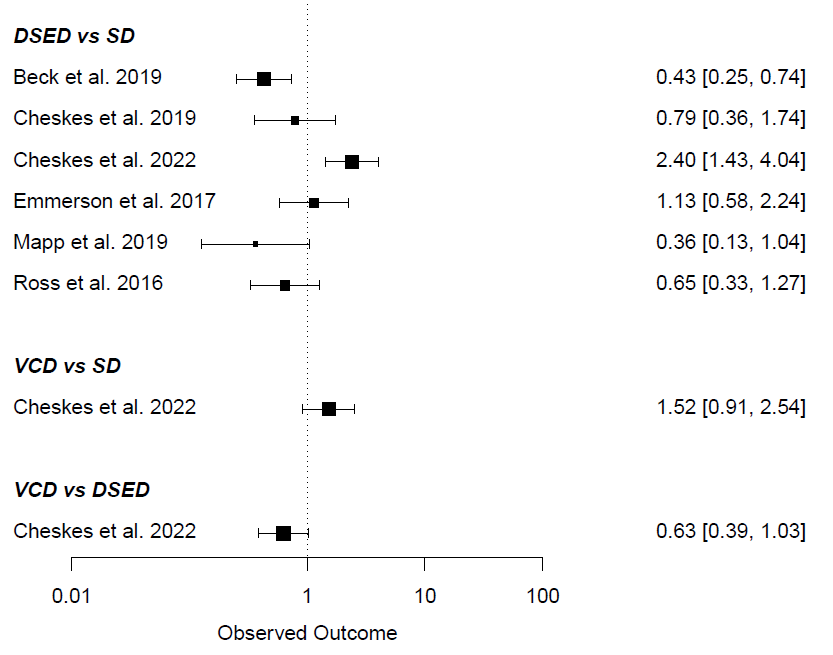


Figure S6: Forest plot of individual study results grouped by treatment component for ROSC.
